# Utility of Human Immune Responses to GAS Antigens as a Diagnostic Indicator for ARF: A Systematic Review

**DOI:** 10.3389/fcvm.2021.691646

**Published:** 2021-07-20

**Authors:** M. Taariq Salie, Kimona Rampersadh, Babu Muhamed, Kélin C. Engel, Liesl J. Zühlke, James B. Dale, Mark E. Engel

**Affiliations:** ^1^Department of Medicine, Faculty of Health Sciences, University of Cape Town, Cape Town, South Africa; ^2^Division of Cardiology, Children's National Health System, Washington, DC, United States; ^3^Children's Heart Disease Research Unit, Department of Paediatrics, University of Cape Town, Cape Town, South Africa; ^4^Division of Infectious Diseases, University of Tennessee Health Science Center (UTHSC), Memphis, TN, United States

**Keywords:** GAS antigens, anti-streptolysin-O, anti-DNase B, ARF diagnosis, systematic review

## Abstract

**Background:** Previous studies have established that streptococcal antibody titer is correlated with a diagnosis of acute rheumatic fever (ARF). However, results vary in the usefulness of GAS antibodies, particularly anti-streptolysin-O (ASO) and anti-DNase B, in confirming a recent GAS infection. Therefore, we sought to provide, from published studies, an evidence-based synthesis of the correlation of streptococcal serology to establish the usefulness of immunological data in aiding the diagnosis of ARF. These findings are anticipated to have implications where echocardiography is not freely available, especially where ARF is rampant.

**Methods:** We conducted a comprehensive search across a number of databases. Applying a priori criteria, we selected articles reporting on studies, regardless of study design, that evaluate the levels of antibodies against GAS-specific antigens in ARF subjects against control values or a published standard. Data were extracted onto data extraction forms, captured electronically, and analyzed using Stata software. Risk of bias was assessed in included studies using the Newcastle-Ottawa Scale (NOS).

**Results and Conclusion:** The search strategy yielded 534 studies, from which 24 met the inclusion criteria, reporting on evaluation of titers for SLO (*n* = 10), DNase B (*n* = 9), anti-streptokinase (ASK) (*n* = 3) amongst others. Elevation in titers was determined by comparison with controls and upper limit of normal (ULN) antibody values as determined in healthy individuals. Meta-analysis of case-controlled studies revealed moderate odds ratio (OR) correlations between ARF diagnosis and elevated titers for SLO (OR = 10.57; 95% CI, 3.36–33.29; 10 studies) and DNAse B (OR = 6.97; 95% CI, 2.99–16.27; 7 studies). While providing support for incorporating SLO and DNase B in the diagnosis of ARF, we present the following reflections: an elevation in SLO and DNase B levels are not consistently associated with an ARF diagnosis; increasing the number of GAS proteins in the test is warranted to improve sensitivity; paired (acute and convalescent) samples could provide a more accurate indication of a rising titer. Use of community-based controls as a standard is not a reliable marker by which to gauge recent GAS infection.

## Introduction

Acute rheumatic fever (ARF), which develops within 2–6 weeks after a preceding non-invasive group A streptococcal (GAS) infection such as streptococcal pharyngitis or scarlet fever, affects ~300,000–500,000 people across the globe each year, the majority of whom live in developing countries. ARF symptoms include fever, arthritis, carditis, rash (erythema marginatum), subcutaneous nodules, and/or Sydenham's chorea (1–3). Since these symptoms are related to other diseases, the Jones criteria has been used since 1944 as a clinical standard in the diagnosis of ARF and rheumatic heart disease (RHD); amongst its criteria, is laboratory evidence of recent streptococcal infection, either through culture or an elevation in serum streptococcal antibodies (3). An accurate diagnosis of ARF ensures proper treatment and reduces the risk of recurrent disease and the development of rheumatic heart disease (4). ARF is often underdiagnosed, by as much as 50%, as reported in a Fijian hospital-based study which compared clinical data against primary care records from health care clinics (5). Okello similarly reports a likelihood of under-representation of the actual number of cases presenting to primary care in Uganda (6), thus highlighting the need to develop simple and practical approaches to diagnosing ARF in primary care in low-resource settings.

The Jones criteria (7) has recently been updated by the American Heart Association (AHA) to suit all types of populations with appropriate recommendations (3, 8). With recent advancements in medical technologies such as echocardiography and Doppler flow assessments, analyzing images of the heart valves has been made easier, with clinicians following the guidelines as described in the Jones criteria (3). However, these tools may not be readily available in areas where ARF is rampant. The Jones criteria has guidelines as to assess the remaining major clinical manifestations which, however, may be difficult to evaluate as these symptoms could be mistaken for or attributed to other illnesses. Symptoms of arthritis may be found in septic arthritis, juvenile idiopathic arthritis, lyme disease, or sickle cell anemia (9). Carditis may be found in mitral valve prolapse, fibroelastoma, cardiomyopathy, or Kawasaki disease. Symptoms associated with chorea may be found in Wilson disease, tic disorder, encephalitis, or other autoimmune diseases such as systemic lupus erythematosus and systemic vasculitis. Therefore, included in the Jones criteria are other mechanisms, such as evidence of a preceding GAS infection to eliminate any doubt in the diagnosis of ARF (9).

The Jones criteria describe evidence of a preceding GAS infection with any one of three scenarios; a positive throat culture for GAS, a positive GAS carbohydrate (GAC) antigen test, or a rise in GAS antibody titers [anti-streptolysin O (ASO) or anti-DNase B] that requires paired samples (at diagnosis and 3 weeks post-suspected diagnosis) (9). These GAS-specific antigens form part of the arsenal of GAS for survival and infiltration of the bacteria into human tissues; thus, these antibodies are present in the sera of GAS-infected individuals (10–12). Streptolysin O (SLO) is a cytolytic toxin released by GAS for cell lysis (13, 14). DNase B is an extracellular virulent protein with DNA-degrading activity (15, 16). These two virulent factors, along with GAC, are mentioned within the Jones criteria; however, there are many other GAS-specific antigens in recent times that have shown potential as putative biomarkers relating to the presence of GAS (17–24).

Few countries have specific guidelines in terms of an ARF diagnosis, specifically including a preceding GAS infection (Supplementary Table 1). We sought, through a comprehensive systematic review of published studies, to conduct an evidence-based synthesis of the utility of streptococcal serology in aiding the diagnosis of ARF. Primarily, our review aimed to assess available published literature regarding the association of antibodies against GAS antigens with ARF. We anticipate that our findings could have implications for the design of future diagnostic tests to confirm recent GAS infection in suspected ARF patients.

## Methods

### Search Strategy and Selection Criteria

According to the Preferred Reporting Items for Systematic Reviews and Meta-Analyses guidelines (25), we performed a systematic literature review from two peer-reviewed databases (PubMed and Scopus) with predefined search terms (Supplementary Table 2). This review asks the following question: What is the utility of GAS serology, specifically SLO and/or DNase B, in providing evidence of a recent GAS infection in diagnosing ARF? In addition, we further sought to explore the potential of other GAS-specific antigens which may provide additional support of a recent GAS infection. The search strategy incorporated both free term text and Medical Subject Headings (MeSH) adapted to suit the particular database. Keywords incorporated a combination of terms relating to group A streptococcus, GAS antigens, SLO, DNase B, serology, immune response, and acute rheumatic fever. Results were complemented by hand searching and citation searches in Google Scholar. The search was not restricted to publication dates or language. Additionally, gray literature including theses and conference proceedings, were also considered for inclusion.

Studies were included if immunological assays were used to evaluate the expression of antibodies evoked by GAS-specific antigens in ARF cases and controls within the same population or the use of a control standard based on the titers of healthy individuals (upper limit of normal, ULN) from the same region. Cases needed to be clinically diagnosed as ARF (peer-reviewed guidelines were not a prerequisite), while controls were documented as those having no history of ARF or RHD. In addition, longitudinal studies evaluating immune responses at more than one time point following new GAS acquisitions were also included. Case reports, narrative reviews, opinion pieces and publications lacking expression data, or referenced methodology and/or accepted guidelines, were excluded from the review. Duplicated studies of datasets and participants were removed, with the final, most recent, publication of the data assessed for inclusion.

### Data Extraction and Article Management

Two reviewers (TS, KR) independently applied the search strategy to the relevant databases. Articles were managed using the Rayyan QCRI web/mobile application (26). Titles and abstracts were evaluated to exclude studies that did not describe the expression of GAS-specific antibodies. Thereafter, full texts of the included titles and abstracts were retrieved and further evaluated against the inclusion criterion (Supplementary Table 3). Rayyan QCRI has a built-in “blind” filter function which prevented the reviewers from observing the other's judgements. Discrepancies were resolved through discussion, involving an arbitrator (third reviewer, ME/BM) where necessary.

Two reviewers (TS, BM) extracted data using a standardized data extraction form and any contradictions were solved through discussion with another of the reviewers (ME). Search results from the databases listed above, published and unpublished studies were managed with Endnote X9 referencing software. Briefly, data extraction consisted of recording the study demographics (number of study participants, geographical region), diagnostic measures, GAS-specific antigens and relevant antibody titer measurements describing elevation. The risk of bias assessment tool (Supplementary Table 4) established by Wells et al. (27) was adapted in questions specific for use in this review, for assessing bias amongst included articles. Using the Newcastle-Ottawa Quality Assessment Scale (NOS) for case-control studies, which were characterized as being of a low or high risk of bias. A study with a low risk of bias is considered to be of high-quality and a low-quality study, with a higher risk of bias. Risk of bias was incorporated into the evaluation of heterogeneity in the pooled analyses.

### Data Analysis

Odds ratio estimates together with their 95% confidence intervals (CIs) were calculated to represent the association between GAS-specific antigens and ARF. The Mantel-Haenszel method was used to pool together odds data from individual studies (28). Variability between studies was evaluated both by assessing forest plots visually, and formally by the heterogeneity tests using χ^2^-based *Q* and *I*^2^ statistic (29). As expected, the studies varied in the constitution of participants and in the types of assays conducted; thus, a random-effects model was used for analysis (30).

We conducted statistical data analyses using Stata version 14.1 (StataCorp, College Station, TX, USA) to estimate the combined effect size (odds ratio and 95% CIs) between GAS antigens and ARF and to generate comparative effect forest plots. Studies were analyzed in subgroups based on the inclusion of an accepted guideline at the time of diagnosis of ARF cases. Where a meta-analysis was not feasible, either because data were too heterogeneous or insufficient to allow for meaningful pooling, we compiled a narrative report of the results. Antigens, for which only a single study was available, thus precluding conducting a meta-analysis, were presented as an odds ratio with its 95% confidence interval (CI). We utilized the respective authors' definition of ULN in defining a rising titer.

## Results

### Overview of Search Strategy and Included Articles

**Figure 1:**
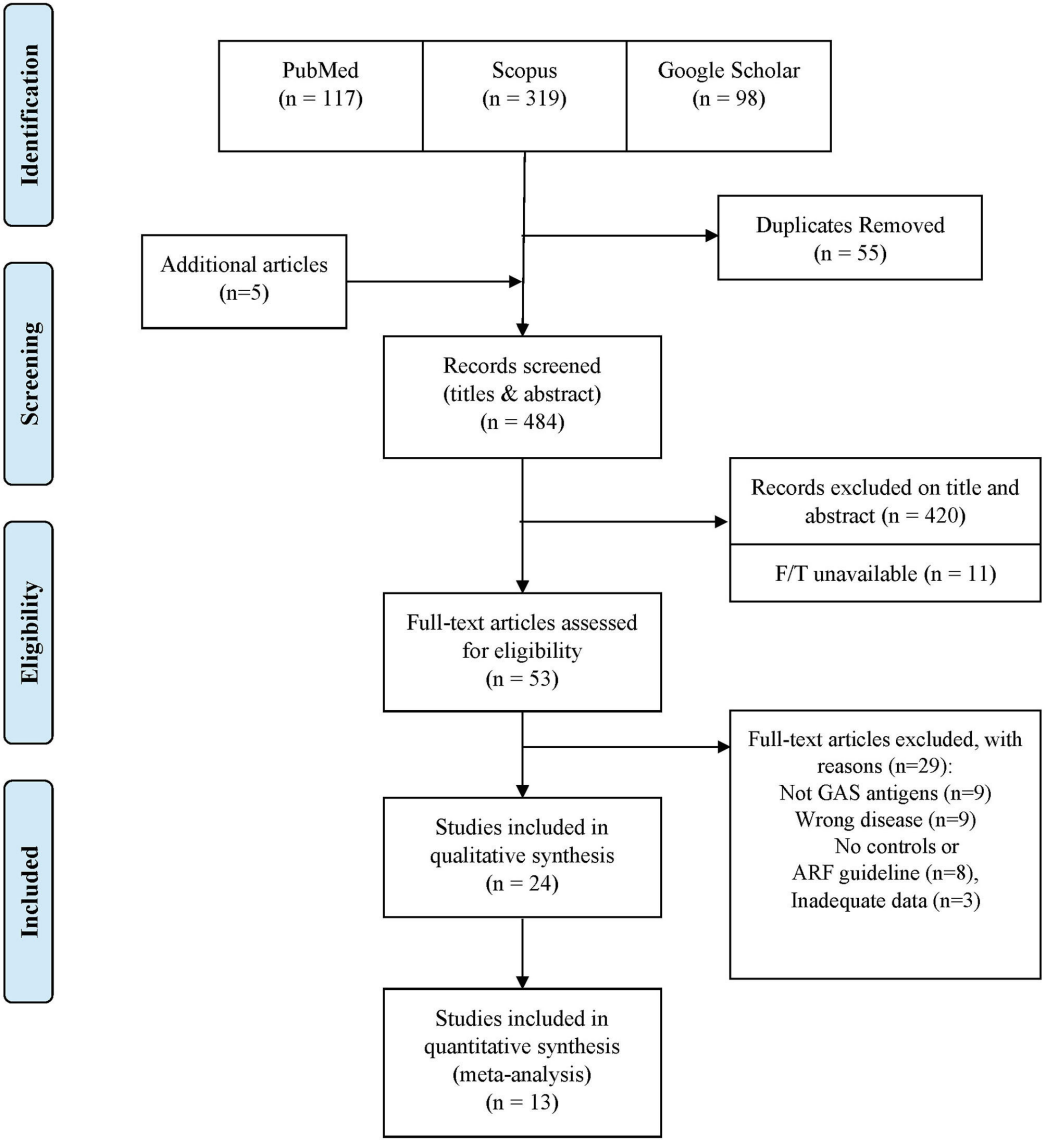
Schematic PRISMA flow diagram of the literature search.

Our search strategy yielded 534 articles which reduced to 479 after excluding duplicates (Figure 1). An additional five studies were included through citation searching, thus leaving 484 for consideration for this review. Following screening of titles and abstracts, 53 articles were deemed potentially relevant and available for full-text evaluation. Twenty-four studies met the inclusion criteria, of which 14 were amenable to meta-analysis. Table 1 shows the characteristics of the included studies. The included articles were published between 1955 and 2020 with sample sizes (cases and controls) ranging from 43 to 2,118 enrolled participants. Studies were conducted in local and university hospitals, clinics, outpatient departments, and schools situated in the study areas. Studies were conducted in the USA (*n* = 7), Japan (*n* = 5), India (*n* = 4), Egypt (*n* = 3), with one article from each of Pakistan, Trinidad, Madagascar, Ethiopia, UK, Australia, and New Zealand. Participants ranged in age from 1 to 89 years. All the articles narrowed their target population to a specific age group, mainly that of children. Only one article (41) made an effort to obtain participants from any age group so as to reflect the national population. A list of the excluded studies with accompanying reasons are detailed in Supplementary Table 5.

**Table 1:** Characteristics of included studies.

**Study ID**	**Country**	**Setting**	**Diagnostic guideline**	**Antigen/Detection method**	**Participants**	**Age**
^a^Ayoub et al. (31)	Grenada/USA	ND	Jones criteria	SLO, DNase B, and GAC—assays described in previous publication (not available)	Grenada: RF (*n* = 32), controls (*n* = 30), Florida: RF (*n* = 32), controls (*n* = 32)	5–32 years
^b^Das et al. (32)	USA/India	ND	NCS	DNase B—ELISA vs. DNA methyl green micromethod	ARF (20), controls (*n* = 20)	ND
^a^Fujikawa and Ohkuni (33)	Japan	ND	RF and RHD Guideline of Japanese Circulation Society	SLO—streptozyme test, DNase B—hemoprobe B test, GAC—ASP kit, SE—enzyme antibody-antigen reaction	RF (*n* = 8), controls (*n* = 354)	6–15 years
^a^Fujikawa and Okuni (34)	Japan	ND	RF and RHD Guideline of Japanese Circulation Society	SLO, DNase B, and SK—multiple enzyme test (streptozyme test)	RF (*n* = 21), controls (*n* = 178)	6–15 years
^a^Fujikawa et al. (35)	Japan	ND	RF and RHD Guideline of Japanese Circulation Society	SLO—described previously, DNase B—hemoprobe B test	RF (*n* = 46), controls (*n* = 278)	3 age groups: <6, 6–16, and >16 years
^b^Gomaa et al. (36)	Egypt	Outpatient RF Clinic	Jones criteria	SLO—turbidimetric immunoassay and ELISA	ARF (*n* = 80), controls (*n* = 80)	ARF-−14.5 years (mean), control-−15.2yrs (mean)
^a^Halbert et al. (37)	USA	ND	NCS	SLO—agar precipitin technique	RF (*n* = 33), non-RF (*n* = 35)	ND
^a^Hanson-Manful et al. (38)	New Zealand	Hospitals	Jones criteria	SLO—turbidimetric technique using SLO kit, DNase B—enzyme inhibition assay, SpnA—bead-based immunoassay	ARF (*n* = 16), controls (*n* = 36)	ARF-−10.6 years (mean), Controls-−6yrs (mean)
^a^Hokonohara et al. (39)	Japan	ND	NCS	SLO—described by other author DNase B—hemoprobe B test, GAC— hemagglutination method	RF (*n* = 28), controls (*n* = NCS)	5–16 years
^d^Hysmith et al. (40)	USA	University associated clinics	–	SLO, DNase B, SCPA, Mrp, J14, SpyCEP, SSE, SOF, SpyAD, and FBP54—ELISA	PIDs (*n* = 41)	6–15 years
^d^Johnson et al. (12)	USA	University associated clinics	–	SLO and DNase B—ELISA	PIDs (*n* = 160)	6–15 years
^a^Julie et al. (41)	Madagascar	Hospital	NCS	SLO—latex agglutination technique	ARF (*n* = 1,690), control (*n* = 428)	1–89 years
^d^Kaplan et al. (42)	USA	NCS	–	SLO, DNase B, and NADase—assays described in previous publication (not available)	PIDs (*n* = 49)	3–6 years
^a^Kawakita et al. (43)	Japan	Elementary school	NCS	SLO—spectrophotometric method, DNase B—micro method, NADase—reduction by alcohol dehydrogenase	ARF (*n* = 3), controls (*n* = 361)	6–11 years
^a^Kotby et al. (44)	Egypt	Hospital	Jones criteria	SLO—rapid latex agglutination	ARF (*n* = 60), controls (*n* = 200)	3 age groups: <6, 6–10, and >10 years
^b^Read et al. (45)	Trinidad	Hospital	Jones criteria	SLO—antibody titre kit	RF (*n* = 44), controls (*n* = 34)	ND
^b^Read et al. (46)	USA	Hospital	Rheumatic Fever Service of The Rockefeller University Hospital	SLO—*in vitro* cellular migration of white blood cells	RF (*n* = NCS), controls (*n* = NCS)	ND
^c^Sagar et al. (47)	India	ND	Jones criteria	SCI, SCPA, and PSA—ELISA	RF (*n* = 24), controls (*n* = 25)	ND
^a^Saini et al. (48)	India	Hospital	Jones criteria	SLO—NCS	ARF (*n* = 26), controls (*n* = 84)	5–15 years
^d^Shet et al. (49)	USA	NCS	–	SLO, DNase B, and SCPA—ELISA	PIDs (*n* = 202)	2–12 years
^b^Tewodros et al. (50)	Ethiopia	ND	NCS	SK—ELISA	ARF (*n* = 11), controls (*n* = 10)	3–12 years
^b^Thakur and Prakash (51)	India	ND	NCS	GAC—ELISA	ARF (*n* = 50), controls (*n* = 50)	ND
^a^Widdowson et al. (52)	UK	Outpatient clinic	NCS	SLO—spectrophotometric method, DNase B—micro method	RF group (*n* = 6), controls (*n* = 44)	16–18 years
^a^Zainab et al. (53)	Pakistan	Hospital	Jones criteria	SLO—kit human tex ASOT	ARF (*n* = 50) (Historic control values)	5–15 years

*ND, no data; NCS, not clearly stated; RF, rheumatic fever; PIDs, participants*.

*SLO, streptolysin O; GAC, group A carbohydrate; SE, streptococcal esterase; SK, streptokinase; SpnA, GAS nuclease A; SCPA, C5a peptidase; Mrp, M-related peptides; J14, C-repeat M peptide; SpyCEP, serine protease; SSE, serine esterase; SOF, serum opacity factor; SpyAD, GAS adhesion and division protein; FBP54, fibronectin-binding protein; SCI, collagen-like surface protein; PSA, putative surface antigen*.

a *Meta-analysis*.

b *Mean titer data*.

c *Single article-antigen*.

d *Longitudinal data*.

The overall quality of studies was moderate, with 12 studies deemed as having a low risk of bias (i.e., a high NOS score; Supplementary Table 4). The included studies clearly described the phenotypes of patients, providing an acceptable case definition and guideline or diagnostic algorithm. Studies diagnosed ARF according to the Jones criteria, with controls examined as having no prior history of ARF or RHD from the same population and age-matched to the cases. Seven of the studies classed as of high risk of bias (NOS <5) were completed before 1990 with authors failing to clearly define the controls or cases with appropriate diagnostic guidelines (33–35, 37, 39, 43, 52). One study Zainab et al. (53) recruited no controls but instead used an ULN cut-off published previously from within the same region.

### Association of SLO Antibody With ARF

**Figure 2:**
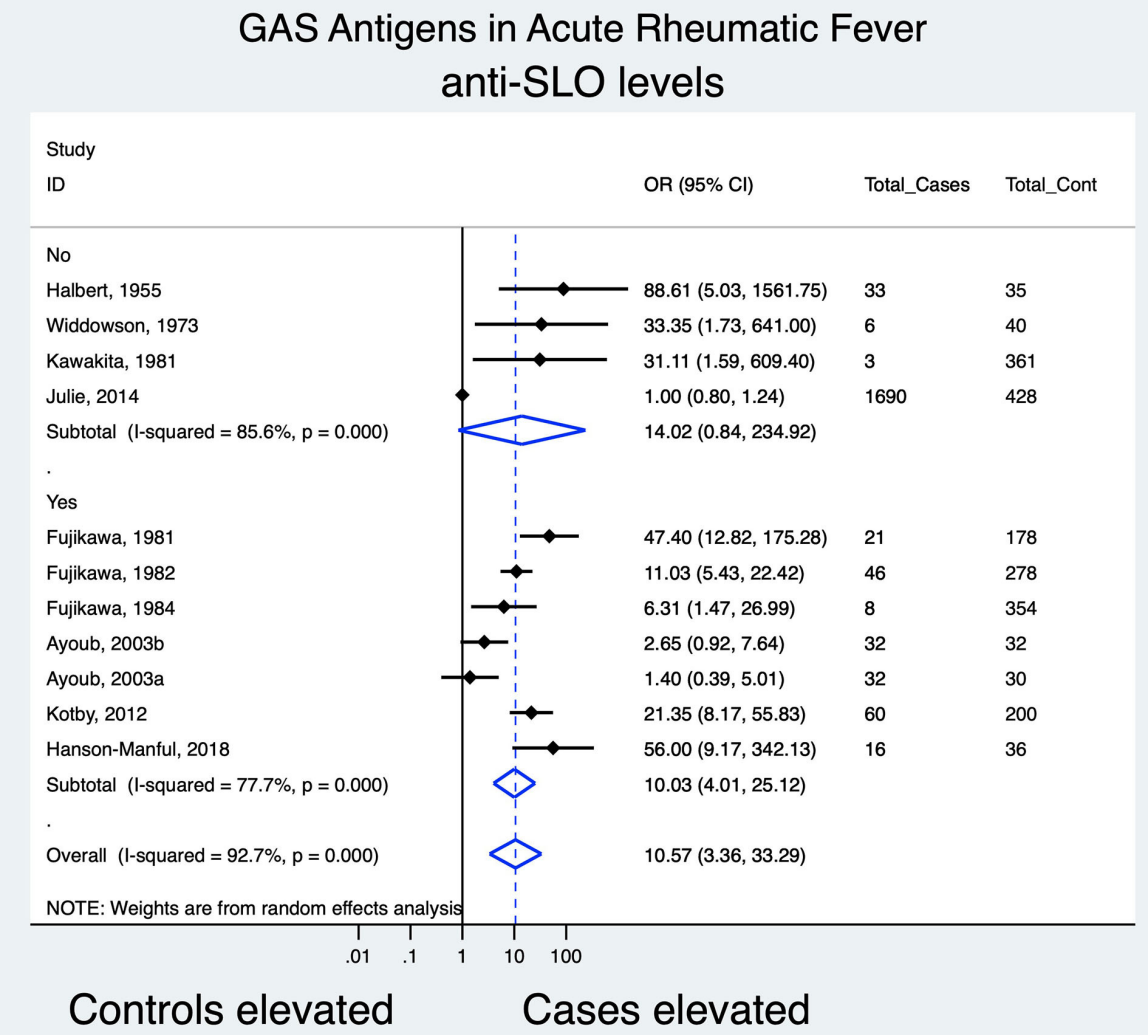
Forest plot evaluating the odds of association between anti-SLO titers and ARF; subgrouping based on whether a guideline was used to diagnose ARF cases.

Ten studies (11 populations; controls, *n* = 1,972; cases, *n* = 1,947) providing data on Anti-SLO titers were amenable to meta-analysis (Figure 2).

Overall, ARF cases showed a greater association of an SLO antibody rise in comparison to controls [odds ratio (OR), 10.57 (95% CI [3.36, 33.29]; (*I*^2^, 77.7%))]. In a subgroup analysis according to whether a guideline for the diagnosis of ARF was used or not, the association was not found to be statistically significant among studies not utilizing a guideline. Amongst studies not included in the meta-analysis due to the absence of a control group, each report a rise in anti-SLO titer: Zainab (53) in 24 of 50 cases (48%) of ARF diagnosed according to the Jones criteria (published standard = 200 IU/ml), Saini (48) in 15 of 26 (58%) cases of ARF as per the Jones criteria guideline (published standard = 262 IU/ml), Hokonohara (39) in 24 of 67 cases of ARF (36%) against published standard = 240 IU/ml.

### Association of DNase B Antibody With ARF

**Figure 3:**
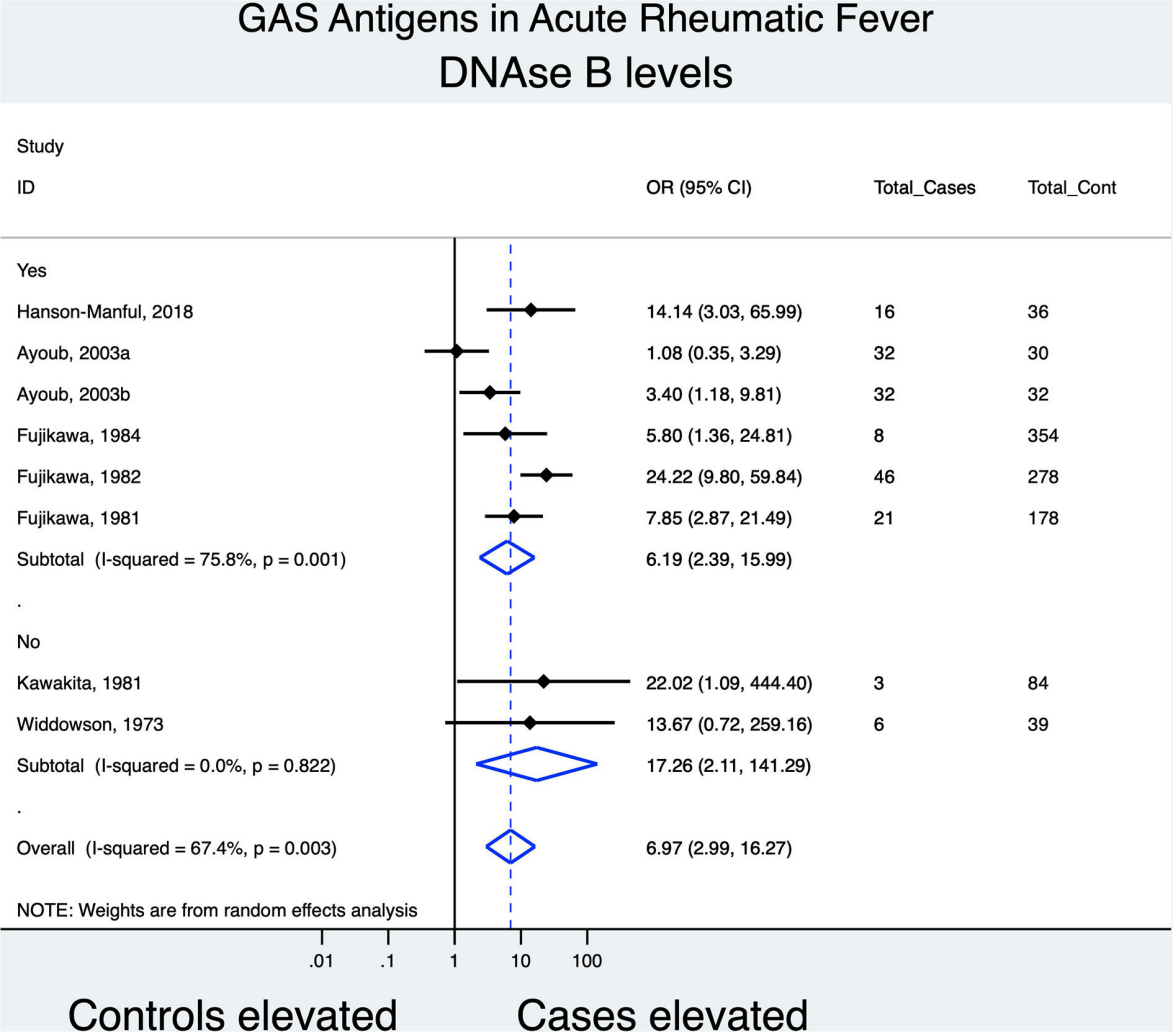
Forest plot evaluating the odds of association between anti-DNase B titers and ARF; subgrouping based on whether a guideline was used to diagnose ARF cases.

Nine studies provided data on Anti-DNase B titers, of which seven (cases, *n* = 164, controls, *n* = 1,031) were amenable to meta-analysis (Figure 3). DNase B antibody levels were significantly increased in ARF cases [OR, 6.97 (95% CI [2.99, 16.27]; (*I*^2^, 67.4%))] in comparison to controls. This result was consistent across all studies, irrespective of the use of a guideline in diagnosing ARF cases. The two studies not included in the meta-analysis did not have control groups as a comparator. Saini (48), used a published standard of 134 IU/ml and reported an elevation of anti-DNase B titers in 85% (22 of 26) of cases while

Hokonohara (39), had 41 cases of ARF and showed a 60% (*n* = 25) titer elevation.

A sensitivity analysis revealed a reduction in the association of anti-SLO levels with ARF in studies with low-risk scores for bias [OR, 4.87 (95% CI, 1.07–22.17); *I*^2^, 93.1%]. Anti-DNase B meta-analysis comprising studies of a low risk of bias, revealed a non-statistically significant association between antibody titers and ARF.

### Association of Other GAS-Specific Antigens With ARF: Streptokinase and GAS Carbohydrate

**Figure 4:**
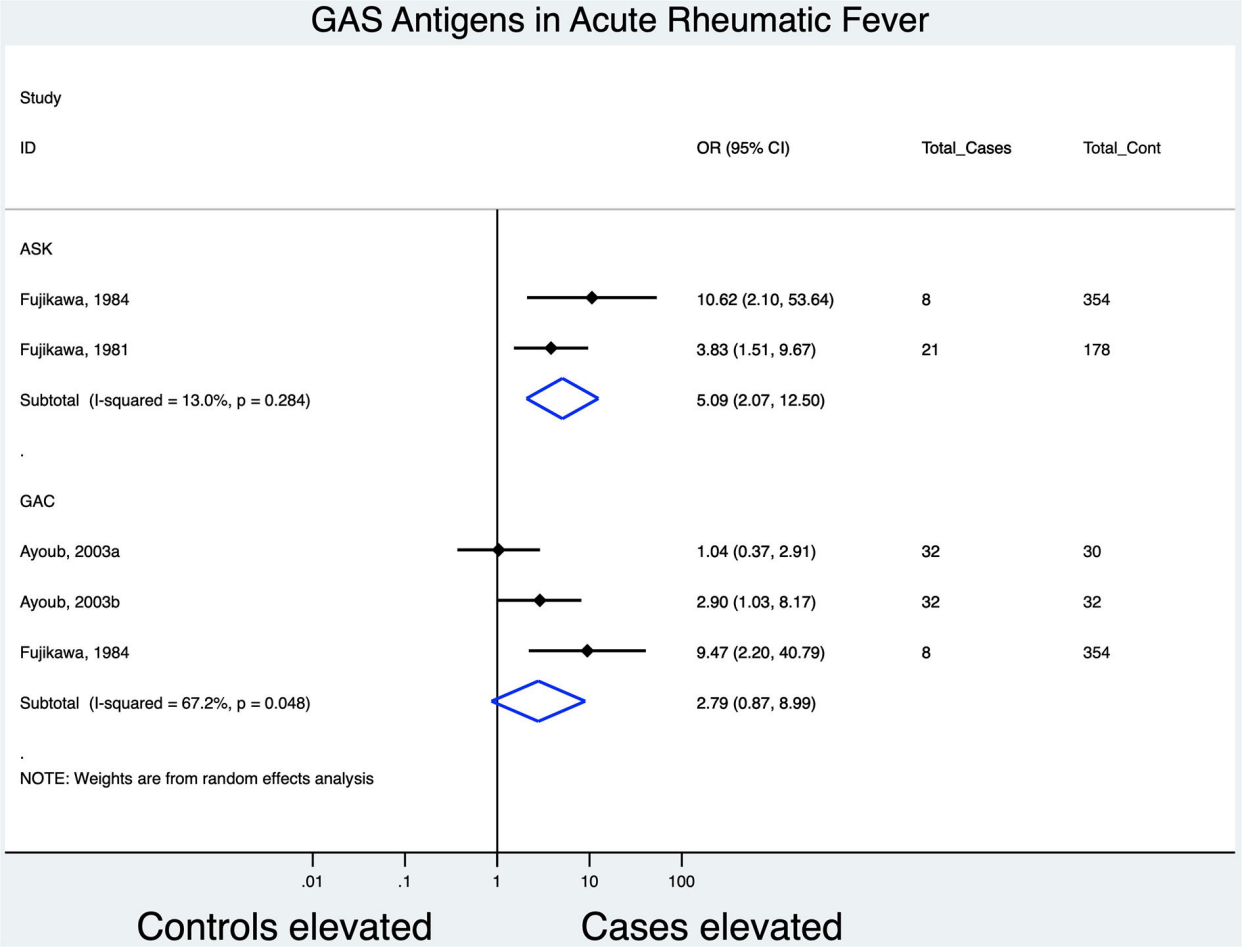
Forest plot evaluating the odds of association between anti-ASK and anti-GAC titers and ARF; subgrouping based on antigen.

Three studies provided data on anti-streptokinase (ASK) titers, of which only two (controls, *n* = 532; cases, *n* = 29) were amenable to meta-analysis (Figure 4) while two studies (controls, *n* = 416; cases, *n* = 72) provided data on Anti-GAC (AGAC) titers (Figure 4).

ASK antibody levels were significantly increased in ARF patients [OR, 5.09 (95% CI [2.07, 12.50], (*I*^2^, 13%))] while GAC responses showed no significance in elevation between cases and controls [OR, 2.79 (95% CI [0.87, 8.99]; (*I*^2^, 67.2%))]. Hokonohara (39), not included in the meta-analysis, showed an elevation of 54% (36 of 67) for ASK and 45% (19 of 41) for AGAC titers in cases of ARF.

### Narrative Review of Studies Not Included in the Meta-Analysis

Four studies (12, 40, 42, 49) reported on the longitudinal assessment of human immune responses to GAS-specific antigens following a new GAS acquisition. Kaplan (42), in evaluating the immune response of 49 participants (aged 3–6 years) against SLO, DNase B, and NADase antigens, showed that GAS pharyngeal-infected participants had an elevated response to GAS antigens compared with participants without infection: SLO, 57% increase vs. 22%, DNase B, 50 vs. 11% and NADase, 43 vs. 22%. Johnson (12) reported on the immune response of 160 participants (aged 6–15 years), from whom 3,491 cultures and 1,679 serum samples were obtained. Over the study period they identified 58 new GAS acquisitions in 45 participants, of which 34.5% (*n* = 20) of the participants showed a significant increase in SLO and DNase B titers. Thirty-six (62.1%) GAS acquisitions were associated with an increase in antibody titer to SLO or DNase B, while 28 showed an increase to SLO and 28 for DNase B. Thus, had only SLO or only DNase B antibody titers been analyzed, eight GAS acquisitions would have been missed. Johnson (12), provided evidence that of 54 serum samples for ASO and 51 samples for anti-DNase B showing an increase in titer following a new GAS acquisition, ~60% were below the ULN described, resulting in the mischaracterization of preceding GAS infections. Furthermore, in amongst GAS carriers, 239 samples had an increased ASO titer above the ULN and 307 samples for anti-DNase B, while only 9.6% (ASO) and 6.5% (anti-DNase B) of these were associated with a true titer increase following a GAS acquisition. It was also shown that in the absence of a culture-positive for GAS, ASO, and anti-DNase B titers were still higher than the ULN for extended periods of time. The study by Hysmith (40) presents the most recent and comprehensive investigation of antibody responses following GAS acquisition. From 41 participants (aged 6–15 years) over a period of 2 years, 51 new GAS acquisitions were documented, with 34 showing an increase in antibody titers against SLO and/or DNase B, illustrating an overall sensitivity of 67% in predicting a new GAS acquisition. Adding increases in antibody levels to GAS SCPA (C5a peptidase) and one additional GAS-shared antigen to SLO and DNase B antibody level increases, improved the overall sensitivity to 76 and 98%, respectively.

Studies that only reported average mean titers were summarized in Supplementary Table 6, provided data were available for SK (50), SLO (36, 45, 46), GAC (51), and DNase B (32). In all the studies, the average mean titers from cases of ARF were considerably higher in comparison to that of the controls.

Studies reporting on less common GAS antigens are summarized in Supplementary Table 7, with the use of GAS-specific antigens: GAS nuclease A (Spn A) (38), collagen-like surface protein (SCI), putative surface antigen (PSA), SCPA (54), streptococcal esterase (SE) (33), Nicotinamide adenine dinucleotidase (NADase) (43), and superoxide dismutase (SOD) (55), in which only Spn A showed a significant OR (95% CI, 56.00 [9.17; 342.13]).

## Discussion

We have presented a comprehensive review of the literature on group A streptococcal antibody responses and their utility in making the clinical diagnosis of ARF. Our meta-analysis provides evidence for a significant association between ARF and anti-SLO, anti-DNase B, and ASK, thus indicating the usefulness of these immunological markers in supporting an ARF diagnosis. This finding is supported by individual studies showing a consistently higher average mean titer in ARF cases over controls. However, there is currently no evidence of an association between GAC antibodies and ARF.

We grouped our findings according to whether peer-reviewed guidelines were used in the clinical diagnosis of ARF. Excluding studies which did not employ a guideline, i.e., those classified as having a higher risk of bias in terms of case definition, reduced the combined estimate of association for anti-SLO and anti-DNase B with ARF from OR 10.57 (95% CI [3.36, 33.29]) to OR 10.03 (95% CI [4.01, 25.12]) and OR 6.97 (95% CI [2.99, 16.27]) to OR 6.19 (95% CI [2.39, 15.99]), respectively. This may indicate caution in terms of investigating only a single antigen in establishing an ARF diagnosis.

Given differences in techniques used to measure antibody titers across the studies, sample size variation, regional differences resulting in the variation of ULN titer levels and published standards used (Table 1), a high degree of heterogeneity was to be expected; hence we employed the random-effect model for the meta-analysis. Sensitivity analyses of studies with a low risk of bias score revealed a reduction in, although still significant, the association of anti-SLO levels with ARF while anti-DNase B analyses showed no statistically significant association between antibody titers and ARF. Unfortunately, the dearth of studies precluded conducting further meaningful subgroup analyses.

We provide a summary of literature meeting our inclusion criteria, but not amenable to meta-analysis through a narrative review. Additional single antigen studies provide further support for the significant association of SK, SLO, GAC, DNase B, Spn A, and SE with ARF but not for SCI, PSA, SCPA, and SOD. For completeness, though not encompassing ARF cases, we included four studies reporting on the longitudinal assessment of human immune response to GAS-specific antigens following a new GAS acquisition in acute and convalescent samples. The studies provided meaningful data in terms of the effectiveness of SLO, DNase B, NADase, and other antigens in detecting a preceding GAS infection. These findings suggest the need to employ an array of antigens to increase the sensitivity of assays confirming a preceding GAS infection, that could be amended within a ARF diagnostic guideline.

Within the studies reporting study limitations, the use of the ULN to describe elevation in titers as evidence of a recent infection risk confounding given its dependence on the controls used within the study. Numerous reports suggest that the ULN of antibody titers against GAS antigens varies with age and geographical location. It has also been reported that titers are lower in adults in comparison with children (56, 57). Johnson (12) and Hysmith (40) identified a number of cases where the participant showed an increase in titer following a new GAS acquisition where the peak titers did not exceed the ULN described for the specific population. Given that cases of GAS carriers demonstrated prolonged elevated responses that exceeded the ULN, caution is warranted since using ULN solely to describe elevation may result in false-negative or false-positive GAS-associations. Thus, these studies strongly suggest that evaluating the rise in titer in paired sequential samples as the most effective way in describing a preceding GAS infection.

This systematic review employed rigorous methods as proposed by the Cochrane Collaboration (58) in synthesizing published resources on the utility of GAS serology in confirming a preceding GAS infection. However, the availability of individual patient data would have further enhanced our findings. As is often the case, data were not reported so as to allow inclusion of some studies into the meta-analysis. Also, there remains a lack of studies in this area.

## Conclusion and Future Research

Providing evidence for a preceding GAS infection remains a challenge. Future studies to evaluate serological tests for evidence of a preceding GAS infection should be designed to overcome the major limitations of the existing evidence base. This can be readily accomplished by ensuring a well-defined case definition as in clear symptoms of ARF with paired sequential sampling of the target population. Furthermore, utilizing an array of GAS antigens is more likely to provide greater sensitivity in providing evidence of a recent infection.

## Data Availability Statement

The original contributions presented in the study are included in the article/Supplementary Material, further inquiries can be directed to the corresponding author/s.

## Author Contributions

MS, BM, and ME were jointly responsible for the conceptualization of the study. MS, KR, BM, and KE contributed to the search strategy and performed data extraction. MS and ME designed and executed the analyses, wrote the first draft, and revised drafts of the manuscript. JD and LZ contributed to the interpretation of the findings. All authors have read and approved the final manuscript.

## Conflict of Interest

The authors declare that the research was conducted in the absence of any commercial or financial relationships that could be construed as a potential conflict of interest.
