# Supplementary material for: Utility of Human Immune Responses to GAS Antigens as a Diagnostic Indicator for ARF: A Systematic Review
Source: Front Cardiovasc Med. 2021 Jul 20;8:691646. doi: 10.3389/fcvm.2021.691646 (PMC8329041; doi:10.3389/fcvm.2021.691646)
Supplement: Supplementary file 1 [file Data_Sheet_1.pdf]

Supplementary Material – Tables and Figures

Table S1. Guidelines associated with the diagnosis of ARF

Lead Author	Publication Title	Country	Describes
Gewitz et al. (2015)	Revision of the Jones Criteria for the Diagnosis of Acute Rheumatic Fever in the Era of Doppler Echocardiography	USA	Diagnostic guidelines (echo) & includes preceding GAS evidence & antibody response
Gerber et al. (2009)	Prevention of rheumatic fever and diagnosis and treatment of acute Streptococcal pharyngitis: a scientific statement from the American Heart Association Rheumatic Fever, Endocarditis, and Kawasaki Disease Committee of the Council on Cardiovascular Disease in the Young, the Interdisciplinary Council on Functional Genomics and Translational Biology, and the Interdisciplinary Council on Quality of Care and Outcomes Research: endorsed by the American Academy of Pediatrics	USA	Diagnostic guidelines & includes preceding GAS evidence & antibody response
Saxena et al. (2008)	Consensus guidelines on pediatric acute rheumatic fever and rheumatic heart disease	India	Diagnostic guidelines
Marzouk et al. (2020)	New guidelines for diagnosis of rheumatic fever; do they apply to all populations?	Egypt	Diagnostic guidelines & antibody responses
Dajani et al. (1993)	Guidelines for the diagnosis of rheumatic fever. Jones Criteria, 1992 update. Special Writing Group of the Committee on Rheumatic Fever, Endocarditis, and Kawasaki Disease of the Council on Cardiovascular Disease in the Young of the American Heart Association	USA	Diagnostic guidelines (JC)
Atatoa-Carr et al. (2008)	Rheumatic fever diagnosis, management, and secondary prevention: A New Zealand guideline	New Zealand	Includes preceding GAS evidence & antibody response
Jack et al. (2019)	Streptococcal Serology in Acute Rheumatic Fever Patients: Findings From 2 High-income, High-burden Settings	New Zealand / Australia	ULN described for SLO and DNase B titres
Khriesat et al. (2003)	Acute rheumatic fever in Jordanian children	Jordan	Assessing guidelines & antibody responses (SLO)

Wilson et al. (2013)	New Zealand guidelines for the diagnosis of acute rheumatic fever: small increase in the incidence of definite cases compared to the American Heart Association Jones criteria	New Zealand	Diagnostic comparison of guidelines (NZ vs JC)
Ralph et al. (2006)	The challenge of acute rheumatic fever diagnosis in a high-incidence population: a prospective study and proposed guidelines for diagnosis in Australia's Northern Territory	Australia	Diagnostic assessment of JC
Carapetis et al. (2007)	An Australian guideline for rheumatic fever and rheumatic heart disease: an abridged outline	Australia	Diagnostic guidelines
Vijayalakshmi et al. (2008)	The efficacy of echocardiographic criterions for the diagnosis of carditis in acute rheumatic fever	India	Diagnostic assessment (echo)
Sanyahumbi et al. (2019)	Two-year evolution of latent rheumatic heart disease in Malawi	Malawi	Diagnostic assessment (echo)
Shiffman (1995)	Guideline maintenance and revision. 50 years of the Jones criteria for diagnosis of rheumatic fever	-	Revisions of JC
Clark et al. (2016)	Using a Low-Risk Population to Estimate the Specificity of the World Heart Federation Criteria for the Diagnosis of Rheumatic Heart Disease	Columbia	Diagnostic assessment (WHF)
Kumar et al. (2016)	Evaluation of the American Heart Association 2015 revised Jones criteria versus existing guidelines	India	Diagnostic comparison of guidelines (IND vs JC)
Alqanatish et al. (2019)	Acute rheumatic fever diagnosis and management: Review of the global implications of the new revised diagnostic criteria with a focus on Saudi Arabia	Saudi Arabia	Implication of revised JC
Boyarchuk, Boytsanyuk & Hariyan (2017)	Acute rheumatic fever: clinical profile in children in western Ukraine	Ukraine	Diagnostic assessment of JC
Pereira et al. (2007)	Jones criteria and underdiagnosis of rheumatic fever	Brazil	Diagnostic assessment of JC
Grassi et al. (2009)	Clinical characteristics and cardiac outcome of acute rheumatic fever in Italy in the last 15 years	Italy	Diagnostic assessment of JC
Olgunturk et al. (2006)	Review of 609 patients with rheumatic fever in terms of revised and updated Jones criteria	Turkey	Diagnostic assessment of JC
Chen et al. (2009)	Changes of manifestations of 122 patients with rheumatic fever in South China during last decade	China	Diagnostic comparison of guidelines (WHO vs JC)
Remenyi et al. (2013)	World Heart Federation criteria for echocardiographic diagnosis of rheumatic heart disease—an evidence-based guideline	Global	Diagnostic guidelines (echo)

Table S2. Search strategy with MeSH terms used for Databases (PubMed, Scopus & Google Scholar):

Subject	MeSH terms
Group A Streptococcus	"Group A Streptococcus" OR "Group A β-haemolytic Streptococcus" OR "Streptococcus pyogenes" OR "GAS" OR "Strep A"
Antigens	"SLO" OR "Streptolysin O" OR "DNase B" OR "SCPA" OR "C5A peptidase" OR "M protein" OR "Streptococcal antigens" OR "GAS antigens" OR "common antigens"
Immunology	"Antibod*" OR "immune response" OR "immunological response" OR "ELISA"
Infection: Post-sequelae	"Rheumatic fever" OR "Acute rheumatic fever" OR "ARF" OR "Rheumatic heart disease" OR "RHD"

Table S3. Inclusion criteria:

**Inclusion criteria**	
Study aims	Studies that describes the expression of bactericidal antibodies evoked by GAS-specific antigens
Detection method	All study designs in which an appropriate immunological assay was completed using sera of participants, producing an upper limit of normal (ULN) to describe elevation in both cases and controls
Participants	All participants with post-sequelae diseases, ARF & RHD with appropriate guidelines and use of an echo completed with controls within the same population and longitudinal studies assessing patients with new GAS acquisitions
Region	Any region
Publication types	Published and unpublished studies
Language	All, with full English abstracts

Table S4. Summary of risk of bias assessment in case–control studies included in the systematic review (Wells et al., 2000)

Study	A				B		C			Quality score
	A1	A2	A3	A4	B1	B2	C1	C2	C3	
Saini et al. (2019)	1	1	1	1	1	1	1	1	1	9
Kotby, Habeeb & Ezz El Arab (2012)	1	1	1	1	1	1	1	1	1	9
Ayoub et al. (2003)	1	1	0	1	1	1	1	1	1	8
Gomaa et al. (2018)[c]	1	1	0	0	1	1	1	1	1	7
Hanson-Manful et al. (2018)	1	1	0	1	0	1	1	1	1	7
Julie et al. (2014)	1	1	1	1	0	0	0	1	1	6
Read, Stanley E et al. (1974)[bc]	1	1	0	0	1	0	1	1	1	6
Read, SE et al. (1986)[bc]	1	1	0	0	1	0	1	1	1	6
Das et al. (2017)[c]	1	1	0	0	0	0	1	1	1	5
Sagar et al. (2012)	1	1	0	0	0	0	1	1	1	5
Tewodros, Norgren & Kronvall (1995)[c]	1	1	0	0	1	0	0	1	1	5
Thakur & Prakash (1996)[c]	1	1	0	0	1	0	0	1	1	5
Fujikawa & Ohkuni (1984)[b]	1	0	0	0	0	0	1	1	1	4
Fujikawa & Okuni (1981)[b]	1	0	0	0	0	0	1	1	1	4
Fujikawa et al. (1982)[b]	1	0	0	0	0	0	1	1	1	4
Widdowson et al. (1974)[b]	1	0	0	0	1	0	0	1	1	4
Halbert, Swick & Sonn (1955)	1	0	0	0	1	0	0	1	1	4
Hokonohara, Yoshinaga & Baba (1987)[b]	1	0	0	0	0	0	0	1	1	3
Kawakita et al. (1981)[b]	1	0	0	0	0	0	0	1	1	3
Zainab et al. (2020)[a]	1	0	0	0	0	0	1	0	0	2

A. Selection of the study groups: A1 right case definition (ARF); A2 right controls definition (no prior history of ARF); A3 the representativeness of the cases (general population); A4 the representativeness of controls (general population).

B. Comparability of the groups: B1 control of main confounders (age/ethnicity); B2 control of any additional factor.

C. Ascertainment of exposure: C1 appropriate method of exposure ascertainment (guideline); C2 same method of exposure ascertainment for cases and controls; C3 same non-response rate of case and control groups.

1: study met the criteria; 0: the study did not meet the criteria.

Quality score: < 5 high risk of bias. ≥ 5 low risk of bias

[a], no controls but case measurement data; [b], old article with minimal patient demographics; [c], mean data only

Table S5. Characteristics of excluded studies

Author	Year	Title	Reason for exclusion
Abo-Zenah	2008	The Reactive Nature of Acute Rheumatic Fever: Evidence from Streptococcal Cell Wall Antigen Detection by immunotechnology	No controls
Baba	1973	The effect of Beta-Lipoprotein of Antistreptolysin-O Antibody Titres	Wrong disease
Bisno	1982	Type-Specific Antibodies to Structurally Defined Fragments of Streptococcal M Proteins in Patients with Acute Rheumatic Fever	Not GAS antigens
Blackwell	2005	Antistreptokinase antibodies: implications for thrombolysis in a region with endemic streptococcal infection	Wrong disease
Brandt	2001	Antibody levels to the class I and II epitopes of the M protein and myosin are related to Group A Streptococcal exposure in endemic populations	Not GAS antigens
Carapetis	2001	Rheumatic fever in a high incidence population: the importance of monoarthritis and low grade fever	No controls
Catarino	2021	Ficolin-3 in rheumatic fever and rheumatic heart disease	Not GAS antigens
Ekelund	2005	Variations in emm Type among Group A Streptococcal Isolates Causing Invasive or Non-invasive Infections in a Nationwide Study	Wrong disease
Garcia	2016	Cardiac Myosin Epitopes Recognized by Autoantibody in Acute and Convalescent Rheumatic Fever	Not GAS antigens
Gray	1981	Cellular Immune Responses to Extracellular Streptococcal Products Rheumatic Heart Disease	Wrong disease (RHD)
Gupta	2016	Immune response against M protein conserved region peptides from prevalent group A Streptococcus in a North Indian population	Not GAS antigens
Jones	2000	Reactivity of Rheumatic Fever and Scarlet Fever Patients' Sera with Group A Streptococcal M Protein, Cardiac Myosin, and Cardiac Tropomyosin: a Retrospective Study	Not GAS antigens
Joseph	2017	Immuno-nephelometric determination of group streptococcal anti-streptolysin O titres (ASOT) from dried blood spots: Method for validating a new assay	Wrong disease
Kreikemeyer	2005	Streptococcus pyogenes Collagen Type I-binding Cpa Surface Protein	Wrong disease
Marshall	2015	Group A Streptococcal Carriage and Seroepidemiology in Children up to 10 Years of Age in Australia	Wrong disease
Martins	2008	Comprehensive analysis of antibody responses to streptococcal and tissue antigens in patients with acute rheumatic fever	No controls
McMillan	2004	Immune response to superoxide dismutase in group A Streptococcal infection	Wrong disease (RHD)
Mori	1996	Persistent Elevation of Immunoglobulin G Titer against the CRegion of Recombinant Group A Streptococcal M Protein in Patients with Rheumatic Fever	Not GAS antigens
Oda	1981	Clinical appraisal of the Antideoxyribonuclease-B (ADN-B) by means of Streptonase-B test	No controls / NG
Oner	2016	Parameters indicative of persistence of valvular pathology at initial diagnosis in acute rheumatic carditis: the role of albumin and CD19 expression	No controls
Reid	2002	Postgenomic Analysis of Four Novel Antigens of Group A Streptococcus: Growth Phase-Dependent Gene Transcription and Human Serologic Response	No controls
Steer	2009	Normal Ranges of Streptococcal Antibody Titers Are Similar Whether Streptococci Are Endemic to the Setting or Not	Wrong disease
Wahid	1996	Mitral regurgitation may be related with previous streptococcal infection	Not GAS antigens
Watanabe	1987	Anti-streptopolysaccharide Antibody in Children with Rheumatic Fever and Scarlet Fever	Minimal data
Watanabe	1981	Follow-up study of ASO, ADN-B, and ASK levels in children with Rheumatic fever	No controls / NG
Watanabe	1976	Antihyaluronidase Level in Children with Rheumatic Fever and Other Streptococcal Infection	Testing assay
Watanabe	1979	The Significance of Measurement of Anti-Deoxyribonuclease-B in the Patients with Streptococcal Infection	Testing assay
Widdowson	1971	An M-associated protein antigen (MAP) of group a streptococci	Not GAS antigens
Zegeye	2016	Throat culture positivity rate and antibiotic susceptibility pattern of beta-hemolytic streptococci in children on secondary prophylaxis for rheumatic heart disease	No controls

Table S6. Evaluation of studies reporting average mean titres in ARF

Study ID	GAS antigen	No. controls	Control mean	No. cases	Cases mean
Tewodros, 1995	SK	10	87.99 OD450nm	11	131.5 OD450nm
Read, 1986[b]	SLO	34	102 todd units	44	495 todd units
Read, 1974[a]	SLO	NS	600u		800u
Thakur, 1996[a]	GAC	50	0.26u	50	0.63
Das, 2017	DNase B	20	SEM: 20.4 +- 6.36ug/ml	NS	SEM: 93.5 +- 22.73ug/ml
Gomaa, 2018	SLO	80	MED: 29.0 UI/ml (11.3–97.0)	80	MED: 116 UI/ml (26.3–172)

[a], Measurement units not given; [b], geometric mean; NS, Not stated; SEM, standard error of mean; MED, median (interquartile range)

Table S7. Evaluation of studies reporting on less common GAS antigens

Study ID	GAS antigen	No. controls	No. cases	ULN	Odds Ratio (95% CI)
Hanson-Manful, 2018	GAS nuclease A (Spn A)	4/36	14/16	170µg/ml	56.00 (9.17; 342.13)
Sagar, 2012	collagen-like surface protein (SCI)	7/25	8/24	0.3 OD405nm	1.29 (0.38; 4.34)
Sagar, 2012	putative surface antigen (PSA)	11/25	13/24	1.4 OD405nm	1.50 (0.49; 4.64)
Sagar, 2012	C5a peptidase (SCPA)	11/25	11/24	1.2 OD405nm	1.08 (0.35; 3.32)
Fujikawa, 1984	streptococcal esterase (SE)	84/354	5/8	400units	5.36 (1.25; 22.89)
Kawakita, 1981	Nicotinamide adenine dinucleotidase (NADase)	3/84	0/3	333U/ml	-
McMillan, 2004	superoxide dismutase (SOD)	4/23	6/23	0.18 OD450nm	1.68 (0.40; 6.97)

References

Alqanatish, J., Alfadhel, A., Albelali, A. & Alqahtani, D. 2019. Acute rheumatic fever diagnosis and management: Review of the global implications of the new revised diagnostic criteria with a focus on Saudi Arabia. *Journal of the Saudi Heart Association.* 31(4):273-281.

Atatoa-Carr, P., Lennon, D., Wilson, N. & Group, N.Z.R.F.G.W. 2008. Rheumatic fever diagnosis, management, and secondary prevention: a New Zealand guideline. *The New Zealand Medical Journal (Online).* 121(1271).

Ayoub, E.M., Nelson, B., Shulman, S.T., Barrett, D.J., Campbell, J.D., Armstrong, G., Lovejoy, J., Angoff, G.H. et al. 2003. Group A streptococcal antibodies in subjects with or without rheumatic fever in areas with high or low incidences of rheumatic fever. *Clinical and diagnostic laboratory immunology.* 10(5):886-890.

Boyarchuk, O., Boytsanyuk, S. & Hariyan, T. 2017. Acute rheumatic fever: clinical profile in children in western Ukraine. *Journal of medicine and life.* 10(2):122.

Carapetis, J.R., Brown, A., Wilson, N.J. & Edwards, K.N. 2007. An Australian guideline for rheumatic fever and rheumatic heart disease: an abridged outline. *Medical journal of Australia.* 186(11):581-586.

Chen, L., Xie, X., Gu, J., Xu, L., Yang, X. & Yu, B. 2009. Changes of manifestations of 122 patients with rheumatic fever in South China during last decade. *Rheumatology international.* 30(2):239-243.

Clark, B.C., Krishnan, A., McCarter, R., Scheel, J., Sable, C. & Beaton, A. 2016. Using a low-risk population to estimate the specificity of the World Heart Federation criteria for the diagnosis of rheumatic heart disease. *Journal of the American Society of Echocardiography.* 29(3):253-258.

Dajani, A.S., Ayoub, E., Bierman, F.Z., Bisno, A.L., Denny, F.W., Durack, D., Ferrieri, P., Freed, M. et al. 1993. Guidelines for the diagnosis of rheumatic fever: Jones criteria, updated 1992: special writing group of the committee on rheumatic fever, endocarditis, and Kawasaki disease of the council on cardiovascular disease in the young, American Heart Association. *Circulation.* 87(1).

Das, S., Dileepan, T., Johnson, D., Kaplan, E. & Cleary, P.P. 2017. Enzyme-linked immunosorbent assay for group A Streptococcal anti-DNase B in human sera, using recombinant proteins-Comparison to the DNA methyl green micromethod. *Journal of immunological methods.* 451:111-117.

Fujikawa, S. & Okuni, M. 1981. Diagnosis of Streptococcal Infection: Previous or Recent: Proceedings of the 5th Conference on Prevention for Rheumatic Fever and Rheumatic Heart Disease. *Japanese circulation journal.* 45(12):1382-1383.

Fujikawa, S. & Ohkuni, M. 1984. Clinical Significance of Anti-streptococcal Esterase (ASE) Determination in Rheumatic Fever and Other Streptococcal Diseases: THE 8TH CONFERENCE ON PRVENTION FOR RHEUMATIC FEVER AND RHEUMATIC HEART DISEASE. *Japanese circulation journal.* 48(12):1330-1333.

Fujikawa, S., Kawakita, S., Kosakai, N., Oda, T., Ohkuni, M., Shiokawa, Y., Watanabe, N. & Yamada, T. 1982. Significance of Anti-deoxyribonuclease-B (ADN-B) Determination in Clinical Practice: THE 6th CONFERENCE ON PREVENTION FOR RHEUMATIC FEVER AND RHEUMATIC HEART DISEASE. *Japanese circulation journal.* 46(11):1180-1183.

Gerber, M.A., Baltimore, R.S., Eaton, C.B., Gewitz, M., Rowley, A.H., Shulman, S.T. & Taubert, K.A. 2009. Prevention of rheumatic fever and diagnosis and treatment of acute Streptococcal pharyngitis: a scientific statement from the American Heart Association Rheumatic Fever, Endocarditis, and Kawasaki Disease Committee of the Council on Cardiovascular Disease in the Young, the Interdisciplinary Council on Functional Genomics and Translational Biology, and the Interdisciplinary Council on Quality of Care and Outcomes Research: endorsed by the American Academy of Pediatrics. *Circulation.* 119(11):1541-1551.

Gewitz, M.H., Baltimore, R.S., Tani, L.Y., Sable, C.A., Shulman, S.T., Carapetis, J., Remenyi, B., Taubert, K.A. et al. 2015. Revision of the Jones Criteria for the diagnosis of acute rheumatic fever in the era of Doppler echocardiography: a scientific statement from the American Heart Association. *Circulation.* 131(20):1806-1818.

Gomaa, M.H., Ali, S.S., Fattouh, A.M., Hamza, H.S. & Badr, M.M. 2018. MBL2 gene polymorphism rs1800450 and rheumatic fever with and without rheumatic heart disease: an Egyptian pilot study. *Pediatric Rheumatology.* 16(1):24.
Grassi, A., Fesslova, V., Carnelli, V., Boati, E., Dell'Era, L., Salice, P., Bardare, M. & Corona, F. 2009. Clinical characteristics and cardiac outcome of acute rheumatic fever in Italy in the last 15 years. *Clin Exp Rheumatol.* 27(2):366-372.
Halbert, S.P., Swick, L. & Sonn, C. 1955. The use of precipitin analysis in agar for the study of human streptococcal infections: II. Ouchterlony and Oakley technics. *The Journal of experimental medicine.* 101(5):557-576.
Hanson-Manful, P., Whitcombe, A.L., Young, P.G., Carr, P.E.A., Bell, A., Didsbury, A., Mitchell, E.A., Dunbar, P.R. et al. 2018. The novel Group A Streptococcus antigen SpnA combined with bead-based immunoassay technology improves streptococcal serology for the diagnosis of acute rheumatic fever. *Journal of Infection.* 76(4):361-368.
Hokonohara, M., Yoshinaga, M. & Baba, Y. 1987. Study of Antibody Response to 4 Streptococcal Antigens in Rheumatic Fever and Kawasaki Disease with or without Cardiovascular Lesions: THE 11th CONFERENCE ON PREVENTION FOR RHEUMATIC FEVER AND RHEUMATIC HEART DISEASE. *Japanese circulation journal.* 51(12):1353-1356.
Jack, S., Moreland, N.J., Meagher, J., Fittock, M., Galloway, Y. & Ralph, A.P. 2019. Streptococcal serology in acute rheumatic fever patients: findings from 2 high-income, high-burden settings. *The Pediatric Infectious Disease Journal.* 38(1):e1-e6.
Julie, Z.N., Arivelo, R.Z., Hendriso, R.D., Ramamonjisoa, A. & Andry, R. 2014. Current practice about the evaluation of antibody to streptolysin O (ASO) levels by physicians working in Antananarivo, Madagascar. *African health sciences.* 14(2):384-389.
Kawakita, S., TAKEUCHI, T., INOUE, J., ONISHI, T. & UEMURA, Y. 1981. Infection of Group A Streptococcus and Antibody Response to Extracellular Antigens: Proceedings of the 5th Conference on Prevention for Rheumatic Fever and Rheumatic Heart Disease. *Japanese circulation journal.* 45(12):1384-1390.
Khriesat, I., Najada, A., Al Hakim, F. & Abu Haweleh, A. 2003. Acute rheumatic fever in Jordanian children. *EMHJ-Eastern Mediterranean Health Journal, 9 (5-6), 981-987, 2003.*
Kotby, A.A., Habeeb, N.M. & Ezz El Arab, S. 2012. Antistreptolysin O titer in health and disease: levels and significance. *Pediatric reports.* 4(1):25-29.
Kumar, D., Bhutia, E., Kumar, P., Shankar, B., Juneja, A. & Chandelia, S. 2016. Evaluation of the American Heart Association 2015 revised Jones criteria versus existing guidelines. *Heart Asia.* 8(1):30-35.
Marzouk, P.A.A., Hamza, H., Mosaad, N., Emam, S., Fattouh, A.M. & Hamid, L. 2020. New guidelines for diagnosis of rheumatic fever; do they apply to all populations? *Turkish Journal of Pediatrics.* 62(3).
Olgunturk, R., Canter, B., Tunaoglu, F.S. & Kula, S. 2006. Review of 609 patients with rheumatic fever in terms of revised and updated Jones criteria. *International journal of cardiology.* 112(1):91-98.
Pereira, B.A., da Silva, N.A., Andrade, L.E., Lima, F.S., Gurian, F.C. & de Almeida Netto, J.C. 2007. Jones criteria and underdiagnosis of rheumatic fever. *The Indian Journal of Pediatrics.* 74(2):117-121.
Ralph, A., Jacups, S., McGough, K., McDonald, M. & Currie, B.J. 2006. The challenge of acute rheumatic fever diagnosis in a high-incidence population: a prospective study and proposed guidelines for diagnosis in Australia's Northern Territory. *Heart, Lung and Circulation.* 15(2):113-118.
Read, S., Reid, H., Fischetti, V., Poon-King, T., Ramkissoon, R., McDowell, M. & Zabriskie, J. 1986. Serial studies on the cellular immune response to streptococcal antigens in acute and convalescent rheumatic fever patients in Trinidad. *Journal of clinical immunology.* 6(6):433-441.
Read, S.E., Fischetti, V.A., Utermohlen, V., Falk, R.E. & Zabriskie, J.B. 1974. Cellular Reactivity Studies to Streptococcal Antigens MIGRATION INHIBITION STUDIES IN PATIENTS WITH STREPTOCOCCAL INFECTIONS AND REHEUMATIC FEVER. *The Journal of clinical investigation.* 54(2):439-450.

Remenyi, B., Carapetis, J., Wyber, R., Taubert, K. & Mayosi, B.M. 2013. Position statement of the World Heart Federation on the prevention and control of rheumatic heart disease. *Nature Reviews Cardiology.* 10(5):284.
Sagar, V., Bergmann, R., Nerlich, A., McMillan, D.J., Schmitz, D.P.N. & Chhatwal, G.S. 2012. Variability in the distribution of genes encoding virulence factors and putative extracellular proteins of Streptococcus pyogenes in India, a region with high streptococcal disease burden, and implication for development of a regional multisubunit vaccine. *Clinical and Vaccine Immunology.* 19(11):1818-1825.
Saini, N., Kumar, D., Swarnim, S., Bhatt, D. & Kishore, S. 2019. Comparison of antistreptolysin O and anti-deoxyribonucleic B titers in healthy children to those with acute pharyngitis, acute rheumatic fever, and rheumatic heart disease aged 5–15 years. *Annals of pediatric cardiology.* 12(3):195.
Sanyahumbi, A., Beaton, A., Guffey, D., Hosseinipour, M.C., Karlsten, M., Minard, C.G., Penny, D.J., Sable, C.A. et al. 2019. Two-year evolution of latent rheumatic heart disease in Malawi. *Congenital heart disease.* 14(4):614-618.
Saxena, A., Kumar, R.K., Gera, R., Radhakrishnan, S., Mishra, S. & Ahmed, Z. 2008. Consensus guidelines on pediatric acute rheumatic fever and rheumatic heart disease. *Indian pediatrics.* 45(7):565-573.
Shiffman, R.N. 1995. Guideline maintenance and revision: 50 years of the Jones criteria for diagnosis of rheumatic fever. *Archives of pediatrics & adolescent medicine.* 149(7):727-732.
Tewodros, W., Norgren, M. & Kronvall, G. 1995. Streptokinase activity among group A streptococci in relation to streptokinase genotype, plasminogen binding, and disease manifestations. *Microbial pathogenesis.* 18(1):53-65.
Thakur, A. & Prakash, K. 1996. Detection of antibody to C-carbohydrate of group A streptococci with enzyme-treated whole bacterial cells as antigen for ELISA. *Journal of medical microbiology.* 45(3):214-218.
Vijayalakshmi, I.B., Vishnuprabhu, R.O., Chitra, N., Rajasri, R. & Anuradha, T.V. 2008. The efficacy of echocardiographic criterions for the diagnosis of carditis in acute rheumatic fever. *Cardiology in the Young.* 18(6):586-592.
Wells, G.A., Shea, B., O'Connell, D.a., Peterson, J., Welch, V., Losos, M. & Tugwell, P. 2000. *The Newcastle-Ottawa Scale (NOS) for assessing the quality of nonrandomised studies in meta-analyses.* Oxford.
Widdowson, J.P., Maxted, W., Newrick, C. & Parkin, D. 1974. An outbreak of streptococcal sore throat and rheumatic fever in a Royal Air Force Training camp; significance of serum antibody to M-associated protein. *Epidemiology & Infection.* 72(1):1-12.
Wilson, N.J., Voss, L., Morreau, J., Stewart, J. & Lennon, D. 2013. New Zealand guidelines for the diagnosis of acute rheumatic fever: small increase in the incidence of definite cases compared to the American Heart Association Jones criteria. *The New Zealand Medical Journal (Online).* 126(1379).
Zainab, S., Saleem, N., Manzoor, A., Khaliq, S., Wasim, A., Khaliq, S. & Shehzad, F. 2020. Antistreptolysin O titer and C-reactive protein levels in pediatric patients of acute rheumatic fever. *The Professional Medical Journal.* 27(07):1335-1339.
